# The green tea component (−)-epigallocatechin-3-gallate protects against cytokine-induced epithelial barrier damage in intestinal epithelial cells

**DOI:** 10.3389/fphar.2025.1559812

**Published:** 2025-05-14

**Authors:** Rita Rosenthal, Anne Waesch, Kopano Valerie Masete, Alain S. Massarani, Jörg-Dieter Schulzke, Nina A. Hering

**Affiliations:** ^1^ Department of Gastroenterology, Rheumatology and Infectious Diseases, Clinical Physiology/Nutritional Medicine, Charité – Universitätsmedizin Berlin, Berlin, Germany; ^2^ Department of General and Visceral Surgery, Charité – Universitätsmedizin Berlin, Berlin, Germany

**Keywords:** (−)-epigallocatechin-3-gallate, TNFα, IFNγ, epithelial barrier breakdown, apoptosis, macromolecular flux, tight junction

## Abstract

**Background:**

Green tea consumption is associated with health benefits, which are mainly attributed to its catechins, especially the main catechin (−)-epigallocatechin-3-gallate (EGCG). Among other beneficial effects, EGCG was shown to be protective in inflammatory bowel disease (IBD), a condition associated with barrier dysfunction. To elucidate the mechanisms behind this, the present study analyzed the impact of EGCG on barrier properties and inflammatory cytokine-induced barrier dysfunction in three different intestinal cell models.

**Methods:**

T84 cells served as a colon model, while Caco-2 and 2D-organoids derived from human duodenum biopsies were used as small intestinal models. Epithelial monolayers grown on filter supports were challenged with EGCG and a combination of the two main IBD cytokines, tumor necrosis factor α (TNFα) and interferon γ (IFNγ). Barrier properties were monitored by measuring transepithelial resistance (TER), macromolecule permeability, apoptosis, and tight junction protein expression and localization.

**Results:**

EGCG protected against barrier defects induced by TNFα and IFNγ. The cytokines decreased TER (T84: 11% ± 1% of initial value; Caco-2: 65% ± 2% of initial value; 2D-organoids: 57 ± 8 Ω*cm^2^ versus control 239 ± 29 Ω*cm^2^) which was prevented by 200 µM EGCG (T84: 89% ± 5%; Caco-2: 89% ± 3%; 2D-organoids: 343 ± 24 Ω*cm^2^; in all three models p < 0.001). In parallel, EGCG attenuated the cytokine-induced increase in macromolecular permeability by reducing apoptosis, as shown by reduced caspase-3 cleavage by >50% compared to cytokine-stimulated controls in all three models (p < 0.001). Furthermore, alterations in tight junction protein expression and localization contributed to barrier protection. In the small intestinal models, 200 µM EGCG stabilized barrier function, as demonstrated by an increase in TER (Caco-2: 105% ± 3% versus control 90% ± 3%; 2D-organoids: 182% ± 12% versus control 105% ± 2%, in both models p < 0.001), upregulation of claudin-4 (Caco-2: 140% ± 15%, p < 0.05; 2D-organoids: 115% ± 5%, p < 0.01) and reduced expression of claudin-2 (Caco-2: 75% ± 10%, p < 0.5; 2D-organoids: 66% ± 6%, p < 0.01) and claudin-7 (Caco-2: 64% ± 7%, p < 0.001; 2D-organoids: 65% ± 9%, p < 0.01). In the colon model, EGCG prevented the delocalization of claudin-1 and -5 that was induced by TNFα and IFNγ.

**Conclusion:**

The green tea component EGCG stabilizes the intestinal barrier and protects against barrier dysfunction induced by pro-inflammatory cytokines. These findings highlight the potential of EGCG as a supportive treatment strategy for IBD.

## 1 Introduction

Green tea (*Camellia sinensis*) is one of the most popular beverages worldwide and a large number of studies have revealed health benefits associated with the habitual consumption of green tea (for reviews see (Afzal et al., 2015; Butt and Sultan, 2009; Chakrawarti et al., 2016; Mokra et al., 2022)). The beneficial effects of green tea are mainly attributed to the polyphenolic flavonoids (catechins), which make up 25–35% of the dry weight of the green tea leaves. These catechins include eight flavonoid-type polyphenolic compounds: (+)-catechin (C), (−)-epicatechin (EC), (+)-gallocatechin (GC), (−)-epigallocatechin (EGC), (+)-catechin gallate (CG), (−)-epicatechin gallate (ECG), (+)-gallocatechin gallate (GCG), and (−)-epigallocatechin-3-gallate (EGCG) (Beguin et al., 2014; Sang et al., 2011). Among these, EGCG, a flavonoid-3-ol polyphenol, is the most abundant and is thought to account for approximately 50% of the total polyphenol content in green tea (Graham, 1992; Oliveira et al., 2016). It is non-toxic and has no reported side effects making this natural substance a popular choice for the treatment and prevention of various diseases.

Extensive research on beneficial effects of green tea has been done over the last years, especially on the isolated catechin EGCG which possesses the most important biological activities compared to the other catechins in green tea (Sano et al., 2001) and is therefore the most used to characterize the beneficial effects of green tea (Alam et al., 2024; Butt and Sultan, 2009; Kciuk et al., 2023). These studies mainly based on *in vitro* and animal experiments demonstrated that green tea or EGCG exerts antioxidant, anticancer, antidiabetic, antibacterial, antiviral, and neuroprotective effects as well as affects the immune system (for review see (Afzal et al., 2015; Chowdhury et al., 2016; Mak, 2012; Zhao et al., 2022)). Epidemiological studies on the association between green tea consumption and the risk of developing human cancer have yielded ambiguous results (Kciuk et al., 2023; Shirakami et al., 2012), whereas some clinical studies indicate positive effects of EGCG on diabetes (Zhang et al., 2017), high blood pressure (Brown et al., 2009; Chatree et al., 2021), and obesity (Chen et al., 2016).

Several studies suggest a protective effect of EGCG in inflammatory bowel disease (IBD) (Li and Weigmann, 2023; Wu et al., 2021; Zhang et al., 2024). The two main forms of IBD, ulcerative colitis and Crohn’s disease are characterized by severe inflammation of parts of the gastrointestinal tract. This is linked to intestinal barrier dysfunction, namely increased epithelial cell apoptosis and tight junction (TJ) perturbations, which facilitate the uptake of antigens, toxins or other noxious agents from the luminal content into the circulation. This in turn favors a pro-inflammatory immune response and enhanced cytokine levels. Intensive research over the last years identified proinflammatory cytokines such as tumor necrosis factor-alpha (TNFα) and interferon-gamma (IFNγ) as key players within this process (Hering et al., 2012; Kobayashi et al., 2020; Schulzke et al., 2009). A pilot study showed an increased remission rate after treatment with EGCG in patients with mild to moderate ulcerative colitis (Dryden et al., 2013). Others found EGCG to be anti-inflammatory by influencing the gut microbiota (Wu et al., 2021) or affecting microbial crosstalk within the oral-gut axis (Ge et al., 2024).

Although EGCG has been studied in various diseases, little is known about its impact on physiological barrier function or its potential protective effects on inflammatory barrier perturbations. The present study aimed to answer this question by investigating the impact of EGCG in three different human intestinal cell models grown on filter supports. The colon cancer cell line T84 was used as a colon model, while Caco-2 cells and human intestinal 2D-organoids served as small intestinal models (Beguin et al., 2014; de León-Rodríguez et al., 2019; Devriese et al., 2017; Masete et al., 2025a). Although, the Caco-2 cell line originates from a colorectal adenocarcinoma, it morphologically and functionally resembles the phenotype of distal ileum enterocytes. Organoid cultures were generated from human duodenum biopsies and were used as a non-cancer-derived model of the upper gastrointestinal tract. Barrier properties of epithelial cell monolayers were assessed by measuring transepithelial resistance (TER), permeability, apoptosis inhibition and TJ properties, e.g. TJ protein expression and localization, after treatment with EGCG in combination with TNFα and IFNγ (TNFα+IFNγ).

## 2 Materials and methods

### 2.1 Cell line culture

Experiments were performed using the human carcinoma cell lines T84 (American Type Culture Collection, ATCC CCL-248, Manassas, VA, United States) and Caco-2 (ATCC, HTB-37), both being well established intestinal cell models (Beguin et al., 2014; de León-Rodríguez et al., 2019; Devriese et al., 2017). T84 cells were cultured in DMEM Nutrient Mixture F-12 Ham medium (Sigma-Aldrich, Taufkirchen, Germany) supplemented with 10% fetal calf serum (Thermo Fisher Scientific, Waltham, MA, United States) and 1% penicillin/streptomycin (Sigma-Aldrich). Caco-2 cells were cultured in Minimum Essential Medium Eagle AqmediaTM (Gibco, Thermo Fisher, Waltham, USA) supplemented with 15% fetal calf serum and (Thermo Fisher) and 1% penicillin/streptomycin (Sigma-Aldrich). Cells were cultured at 37°C in a humidified incubator with 5% CO_2_.

For all functional studies, cells were seeded onto Millicell PCF 3.0 μm pore size filter supports with an effective area of 0.6 cm^2^ (Millipore, Schwalbach, Germany) to form polarized epithelial monolayers as described previously (Dias and Yatscoff, 1994; Hidalgo et al., 1989). Ten (T84) or fourteen to fifteen (Caco-2) days old monolayers were used for all experiments.

### 2.2 Organoid culture

The organoid study was performed in compliance with the Ethics Committee of Charité-Universitätsmedizin Berlin (EA4/015/13). Duodenal biopsy samples were obtained from a 32-year-old healthy female who was scheduled to undergo routine endoscopy and gave written informed consent. Three-dimensional (3D) organoids were generated and passaged as previously described (Masete et al., 2025a). 3D organoids were dissociated into single cells using TrypLE™ Express (Gibco/Thermo Fisher Scientific) for 10 min at 37°C. Roughly 5∙10^5^ cells were seeded as 2D organoids (organoid monolayers) on uncoated filter supports (0.6 cm^2^, Millipore). Confluent 21-day-old organoid monolayers were used for experiments.

### 2.3 Experimental set up

EGCG (Sigma Aldrich, Schnelldorf, Germany) was dissolved in dimethyl sulfoxide (DMSO, Sigma-Aldrich) and added to the cell culture medium at 100 or 200 μM, usually on the apical and basolateral side to avoid osmotic effects. Control monolayers were treated with equal amounts of DMSO.


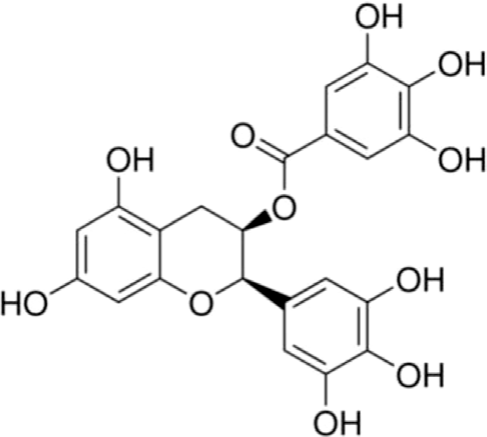


Structure of EGCG (−)-epigallocatechin-3-gallate (https://www.sigmaaldrich.com/DE/de/product/sigma/e4143).

T84 and Caco-2 monolayers were preincubated for 2 hours (h) with EGCG, before the cytokines (25 ng/mL TNFα and 100 ng/mL IFNγ) were added to the basolateral medium. 10 μM of the pan-caspase inhibitor Q-VD-Oph (MedChemExpress/Hycultec, Beutelsbach, Germany) was applied apically and basolaterally 2 h before challenging with cytokines.

Confluent organoid monolayers were pre-stimulated with 1 ng/mL IFNγ for 72 h. Organoid monolayers were preincubated with 200 µM EGCG within the last 18 h of the IFNγ pre-stimulation (overnight). Cytokine challenge was then carried out for up to 10 h with 5 ng/mL TNFα, 1 ng/mL IFNγ and 200 µM EGCG.

Barrier integrity of the monolayers was tested by measuring the transepithelial resistance (TER) with chopstick electrodes, as described previously (Heller et al., 2005). TER was monitored before the addition of EGCG, inhibitors, and cytokines (initial value) and after cytokine challenge at the indicated time points. TER was given in percentage of the initial value, which was set as 100% for T84 and Caco-2 cells. For organoid monolayers, also absolute values were given. Monolayers were then used either for functional studies in Ussing chambers, immunofluorescence staining or Western blot analysis.

### 2.4 Flux measurements in Ussing chambers

Flux measurements were performed in Ussing chambers under voltage-clamp conditions. The standard perfusion solution contained in mM: NaCl, 113.6; Na_2_HPO_4_, 2.4; NaH_2_PO_4_, 0.6; KCl, 5.4; NaHCO_3_, 21; MgCl_2_, 1.2; CaCl_2_, 1.2; Glucose, 10. Dialyzed fluorescein-iso-thio-cyanate dextran 4 kDa (FD4, Sigma–Aldrich, Taufkirchen, Germany) was added to the apical side of the monolayers at a final concentration of 0.2 mM. Samples of 300 µL were collected from the basolateral side in intervals of 20 min and replaced with 300 µL of the perfusion solution, which was corrected for in the calculation. The flux of FD4 from the apical to the basolateral side was very low (<0.1 nmol/h^−1^/cm^−2^), making the change in FD4 concentration in the apical chamber negligible. FD4 fluxes were determined from FD4 concentrations in the samples at six time intervals, which were measured in a plate reader (TECAN infinite M200, Männedorf, Switzerland) at 525 nm. Permeability P (cm·s^−1^) was calculated from the ratio of flux J (mol·h^−1^·cm^−2^) and concentration Δc (mol/L) of the tracers in the Ussing chamber: P = J/Δc.

### 2.5 Western blot analysis

For analysis of TJ protein expression by Western blot, cells were scraped from the filter supports and proteins were extracted using lysis buffer containing 10 mM Tris (pH 7.5), 150 mM NaCl, 0.5% Triton X-100, 0.1% SDS, and complete protease inhibitor mixture (Roche, Mannheim, Germany). To study caspase-3 cleavage by Western blotting, apical cell culture supernatants were collected from incubated cells and centrifuged at 15,000 × g for 5 min. The resulting pellets were lysed together with monolayers in lysis buffer containing 100 mM imidazole, 100 mM KCl, 300 mM sucrose, 2 mM MgCl_2_, 10 mM EGTA, 1 mM NaF, 1 mM NaVO_3_, 1 M Na_2_MO_4_, 0.2% Triton X-100, and complete protease inhibitor. Proteins were separated by SDS page and blotted onto a PVDF membrane, which was incubated with primary and secondary antibodies (Supplementary Table S1). Signal detection was conducted after a 5 min incubation in SuperSignal West Pico PLUS Stable Peroxide Solution (Thermo Fisher Scientific, Waltham, MA, United States), using the Fusion FX7 imaging system. Densitometric analysis was performed using ImageJ (international open-source project). The expression levels of TJ proteins and cleaved caspase-3 were normalized to β-actin as loading control.

### 2.6 Immunofluorescence staining and confocal laser scanning microscopy

For immunofluorescence staining, monolayers on filter inserts were washed with PBS and fixed for 10 min in 4% paraformaldehyde (Thermo Fisher Scientific, Waltham, MA, United States) at room temperature. After washing with PBS and permeabilization with 0.5% Triton X-100, blocking was carried out with a solution containing 5% goat serum, 0.05% Triton X-100, and 1% bovine serum albumin. Incubation with primary antibodies against TJ proteins or full/cleaved caspase-3 (Supplementary Table S1) was done overnight. After rinsing with blocking solution, the membranes were incubated with secondary antibodies conjugated to Alexa-Fluor 488 or 594 for 1 hour (1:200) at 37°C (Supplementary Table S1). Nuclei were counterstained with 4′,6-diamidino-2-phenylindole (DAPI, 1:1000, Roche AG, Mannheim, Germany) for 10 min. Cells on filter inserts were embedded in ProTaqs MountFluor (Biocyc, Luckenwalde, Germany) and visualized using a confocal laser scanning microscope (Zeiss 780, Zeiss, Jena, Germany).

### 2.7 Statistical analysis

Data are shown as means ± SEM (standard error of the mean), n refers to the number of experiments. Statistical analysis was performed using GraphPad/Prism version 9.4.0 (GraphPad Software, Inc. San Diego, CA, United States). Normality of the data was assessed using the Shapiro-Wilk test. Normally distributed data were analyzed using an ordinary one-way ANOVA and Sidak’s multiple comparisons test for comparison of the control group to all other groups and TNFα + IFNγ versus EGCG in combination with TNFα + IFNγ. If the data were not normally distributed, the Kruskal-Wallis test was used. p-values less than 0.05 were considered significant, significance levels are denoted *^, #^ = p < 0.05, **^, ##^ = p < 0.01, ***^, ###^ = p < 0.001 with *^,^ **^,^ *** tested versus control, and ^#, ##, ###^ tested versus TNFα + IFNγ.

## 3 Results

### 3.1 Protective effects of EGCG against TNFα- and IFNγ-induced barrier defects in T84-monolayers

**FIGURE 1:**
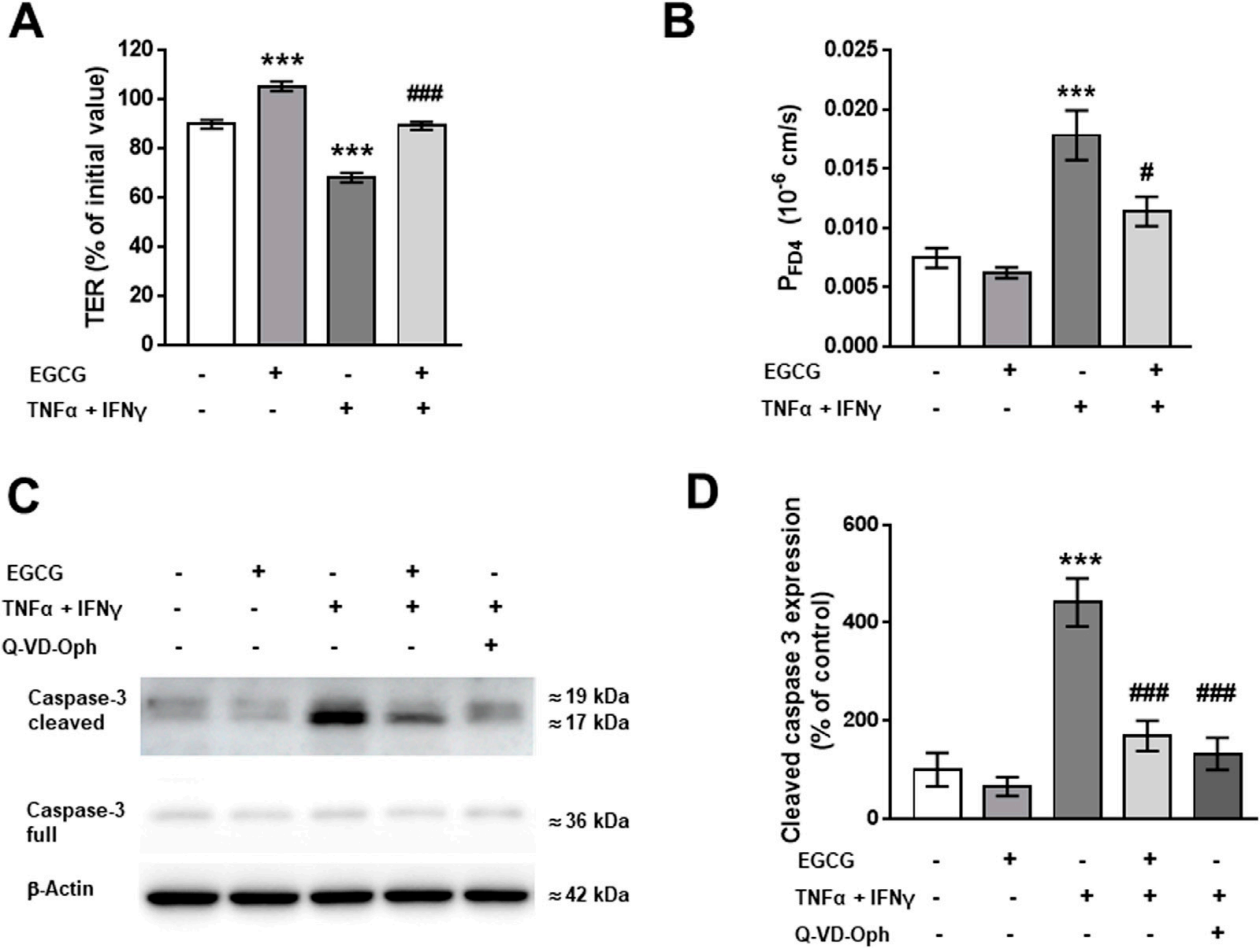
Effects of EGCG on epithelial barrier dysfunction caused by TNFα and IFNγ in T84 monolayers. T84 monolayers were incubated for 2 h with 200 µM EGCG, before TNFα (25 ng/mL) and IFNγ (100 ng/mL) were added. **(A)** Effect of EGCG, applied on both sides of the monolayer, on TER, measured 48 h after cytokine treatment. Values are given in % of initial value, control was set to 100%, n = 15. **(B)** Effect of EGCG on the cytokine-induced increase in FD4 permeability, calculated from transepithelial flux measurements in Ussing chambers, n = 9–24. **(C)** Representative Western blots and **(D)** densitometric analysis of cleaved caspase 3. β-actin was used as loading control and pan-caspase inhibitor Q-VD-Oph served as positive control. Untreated cells set 100%, n = 3–9. ***p < 0.001 vs. control; ^###^p < 0.001 TNFα + IFNγ vs. EGCG + TNFα + IFNγ or Q-VD-Oph + TNFα + IFNγ.

To investigate the effects of EGCG on the barrier integrity of cultured T84 monolayers, TER was measured with chopstick electrodes 48 h after addition of TNFα and IFNγ. As shown in Figure 1A, EGCG did not increase TER significantly, but inhibited the cytokine-induced decrease in TER. The protective effect of EGCG was less pronounced after a shorter incubation time (24 h) and with a lower concentration of EGCG (100 µM) (Supplementary Figure S1). Thus, the concentration of 200 µM EGCG and an incubation time of 48 h were used in all following T84 experiments. Consistant with its protective effect on TER, EGCG inhibited the increase in FD4 permeability (Figure 1B). As it is known that TNFα and IFNγ cause epithelial cell apoptosis (Bruewer et al., 2003; Li et al., 2008), we tested whether the protective effects of EGCG were due to apoptosis inhibition. Western blot analysis showed cytokine-induced caspase-3 cleavage, which was inhibited by EGCG. The caspase 3 inhibitor, Q-VD-Oph, served as a positive control (Figures 1C, D).

**FIGURE 2:**
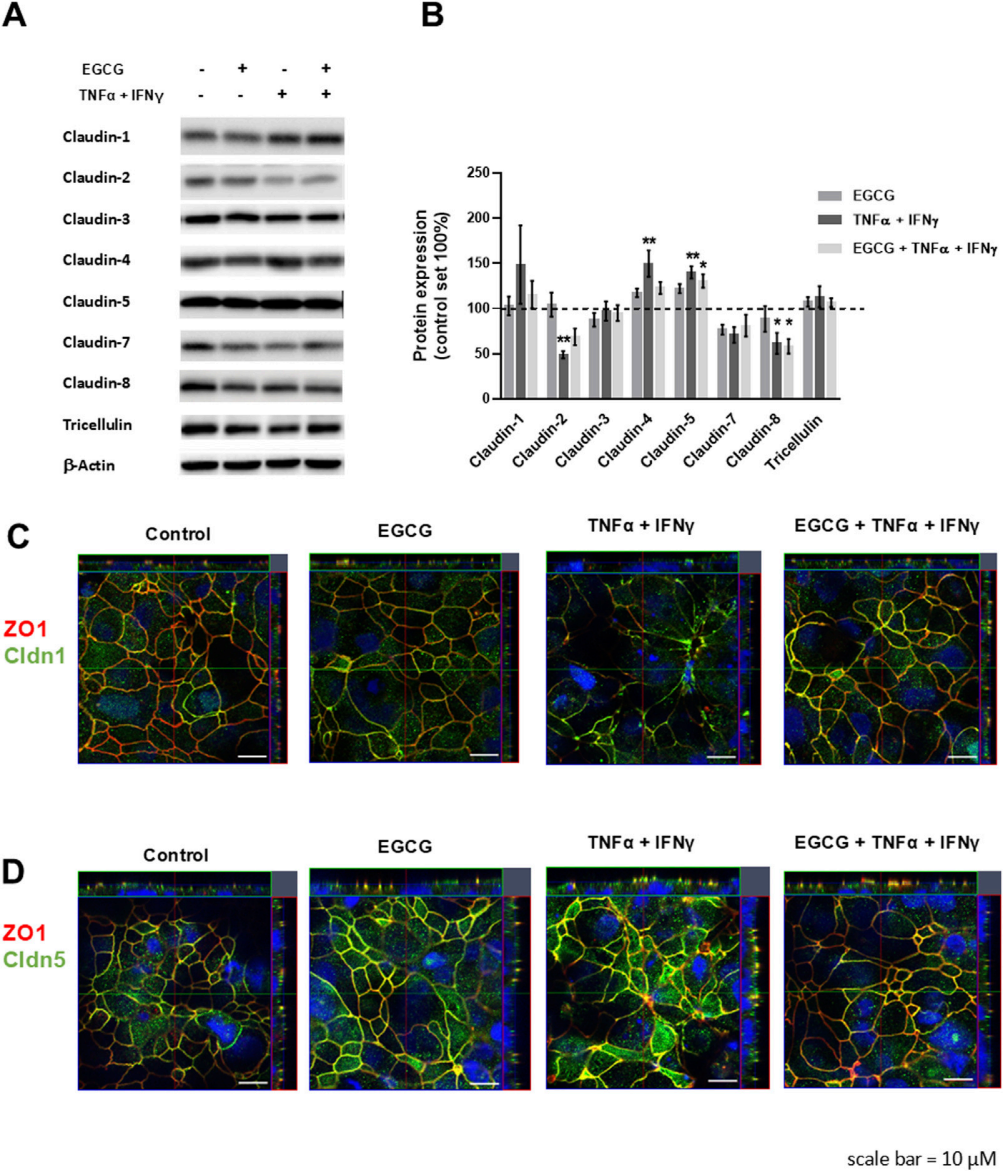
Protective effects of EGCG on TJ protein expression and localization in T84 monolayers. **(A)** Representative Western blots of TJ proteins and **(B)** densitometric analysis under control conditions, incubation with EGCG (200 µM), TNFα (25 ng/mL) + IFNγ (100 ng/mL), and TNFα + IFNγ with EGCG preincubation. Protein expression was normalized to the loading control β-actin and protein expression in untreated cells was set 100% (n = 6–12, *p < 0.05, **p < 0.01). **(C)** Representative immunofluorescence images of claudin-1 (green) and **(D)** claudin-5 (green) in combination with zonula occludens protein-1 (ZO-1, red), obtained in z-stacks using confocal laser-scanning microscopy. Nuclei are DAPI stained (blue). Scale bar represents 10 µm.

TNFα and IFNγ are also known to disturb TJ proteins (Amasheh et al., 2010; Rosenthal et al., 2017). Therefore, the expression and/or subcellular localization of TJ proteins was analyzed under different conditions. As shown in Figures 2A, B, EGCG had no effect on the expression of claudin-1, -2, -3, -4, -5, -7, -8, and tricellulin. Additionally, the TNFα+IFNγ-induced downregulation of claudin-2 and claudin-8 as well as the upregulation of claudin-4 and claudin-5 was not changed by preincubation with EGCG (Figures 2A, B). However, EGCG prevented the delocalization of claudin-1 and -5 that was induced by TNFα+IFNγ. In cytokine-challenged monolayers, claudin-1 was redistributed from the TJ, indicated by the lack of colocalization with the TJ marker ZO-1. This redistribution was inhibited by EGCG (Figure 2C). In addition, the cytokine treatment caused an internalization of claudin-5, which was prevented by EGCG (Figure 2D).

### 3.2 Barrier-stabilizing and protective effects of EGCG against TNFα- and IFNγ-induced barrier defects in Caco-2-monolayers

**FIGURE 3:**
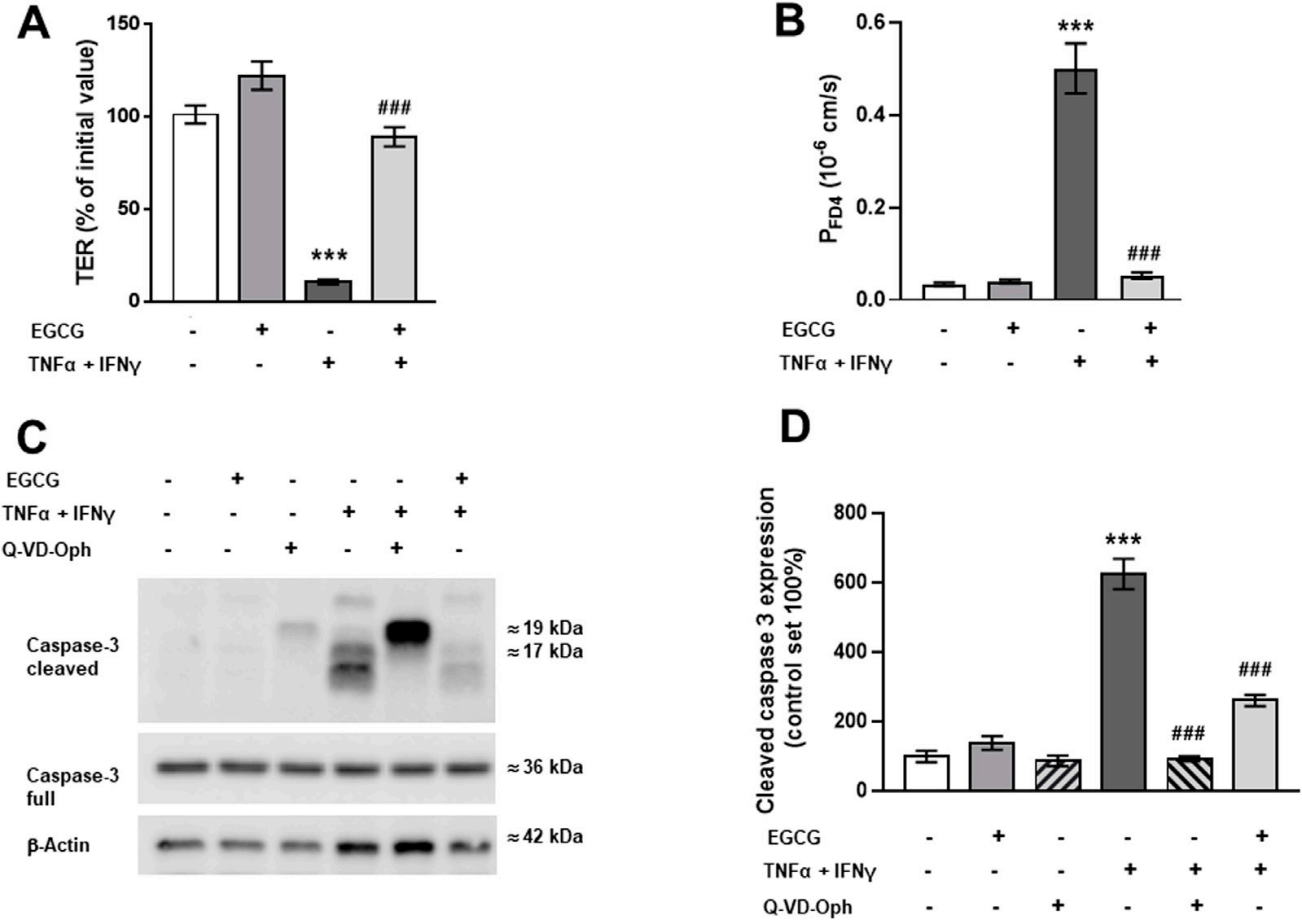
Impact of EGCG on barrier function and cytokine-induced perturbation in Caco-2 monolayers. Caco-2 monolayers were pre-incubated for 2 h with 200 µM EGCG, before TNFα (25 ng/mL) and IFNγ (100 ng/mL) were added. **(A)** Effect of 200 µM EGCG on TER, measured 24 after cytokine treatment. Values are given in % of initial value, control was set to 100% (n = 15). **(B)** Effect of EGCG on the cytokine-induced increase in FD4 permeability, calculated from transepithelial flux measurements in Ussing chambers, measured 24 h after cytokine treatment (n = 3–7). **(C)** Representative Western blots and **(D)** densitometric quantification of cleaved caspase-3 with β-actin as loading control and Q-VD-Oph as positive control (control level set 100%; n = 5). **p < 0.01, ***p < 0.001 vs. control; ^#^p < 0.05, ^###^p < 0.001 TNFα + IFNγ vs. EGCG + TNFα + IFNγ or Q-VD-Oph + TNFα + IFNγ.

In Caco-2 monolayers, EGCG (200 µM) treatment induced an increase in TER of about 15% compared to untreated control monolayers while TNFα+IFNγ caused a TER drop to 68% of the initial value within 24 h (Figure 3A).

As this cytokine effect was not significantly higher after 48 h (Supplementary Figure S2), all further investigations were done after 24 h. EGCG preincubation partially inhibited the cytokine-induced TER decrease (Figure 3A). Similar to the findings in T84 cells, EGCG attenuated the cytokine-induced increase in FD4 permeability (Figure 3B) and blocked the apoptosis caused by TNFα + IFNγ. This was confirmed by reduced caspase-3 cleavage in Western blot analysis (Figures 3C, D) and immunofluorescence staining (Supplementary Figure S3).

**FIGURE 4:**
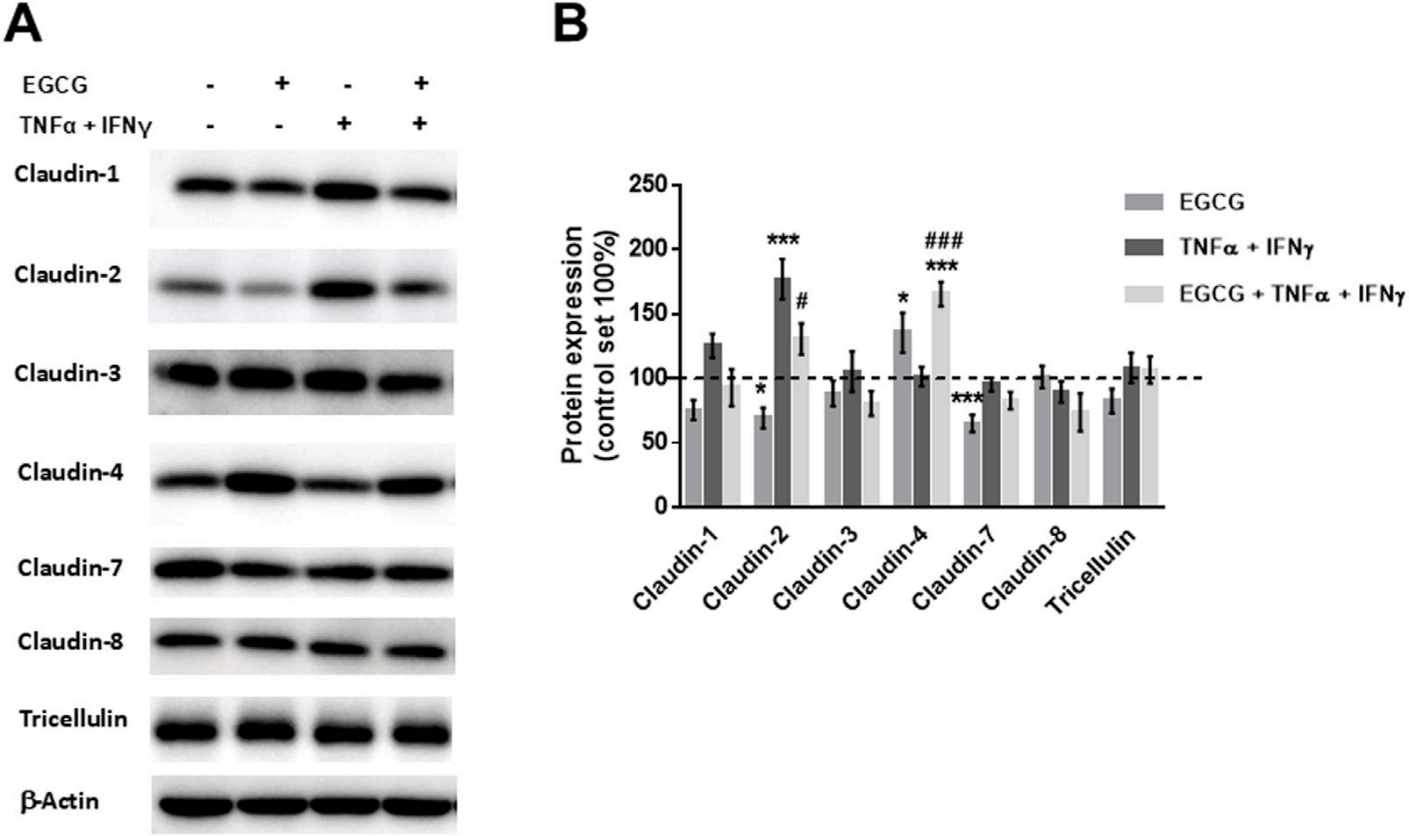
EGCG affects TJ protein expression *per se* and under inflammatory conditions in Caco-2 monolayers. **(A)** Representative Western blots of TJ proteins and **(B)** densitometric analysis under control conditions, incubation with EGCG (200 µM), TNFα (25 ng/mL) + IFNγ (100 ng/mL), and TNFα + IFNγ with EGCG pre-incubation. Protein expression was normalized to the loading control β-actin, protein expression in untreated cells was set 100% (n = 6–12). *p < 0.05, ***p < 0.001 vs. control; ^#^p < 0.05, ^###^p < 0.001 versus TNFα + IFNγ.

TJ protein expression of Caco-2 monolayers showed that EGCG did not affect the expression of claudin-1, -3, -8, and tricellulin, whereas claudin-4 was upregulated and claudin-2 and -7 were downregulated by EGCG. In contrast, TNFα + IFNγ upregulated claudin-2 expression (Figures 4A, B).

### 3.3 Barrier-stabilizing and protective effects of EGCG on human 2D-organoid monolayers

**FIGURE 5:**
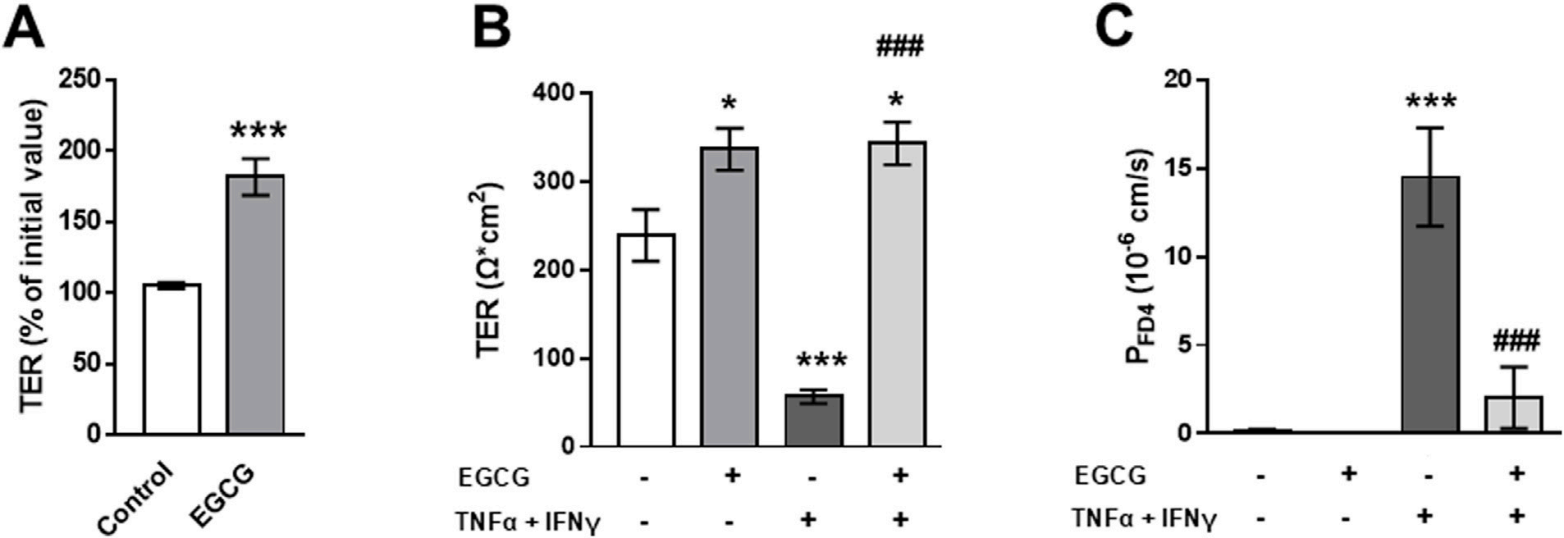
EGCG effects on barrier function and cytokine-induced perturbation in 2D-organoids. **(A)** Organoid monolayers were preincubated with 200 µM EGCG overnight and the effect on TER was monitored with chopstick electrodes. **(B)** Effect of EGCG on cytokine- (5 ng/mL TNFα and 1 ng/mL IFNγ) induced TER decrease and **(C)** increase in FD4 permeability calculated from transepithelial flux measurements in Ussing chambers (n = 4–5). *p < 0.05, ***p < 0.001 vs. control; ^###^p < 0.001 TNFα + IFNγ vs. EGCG + TNFα + IFNγ.

**FIGURE 6:**
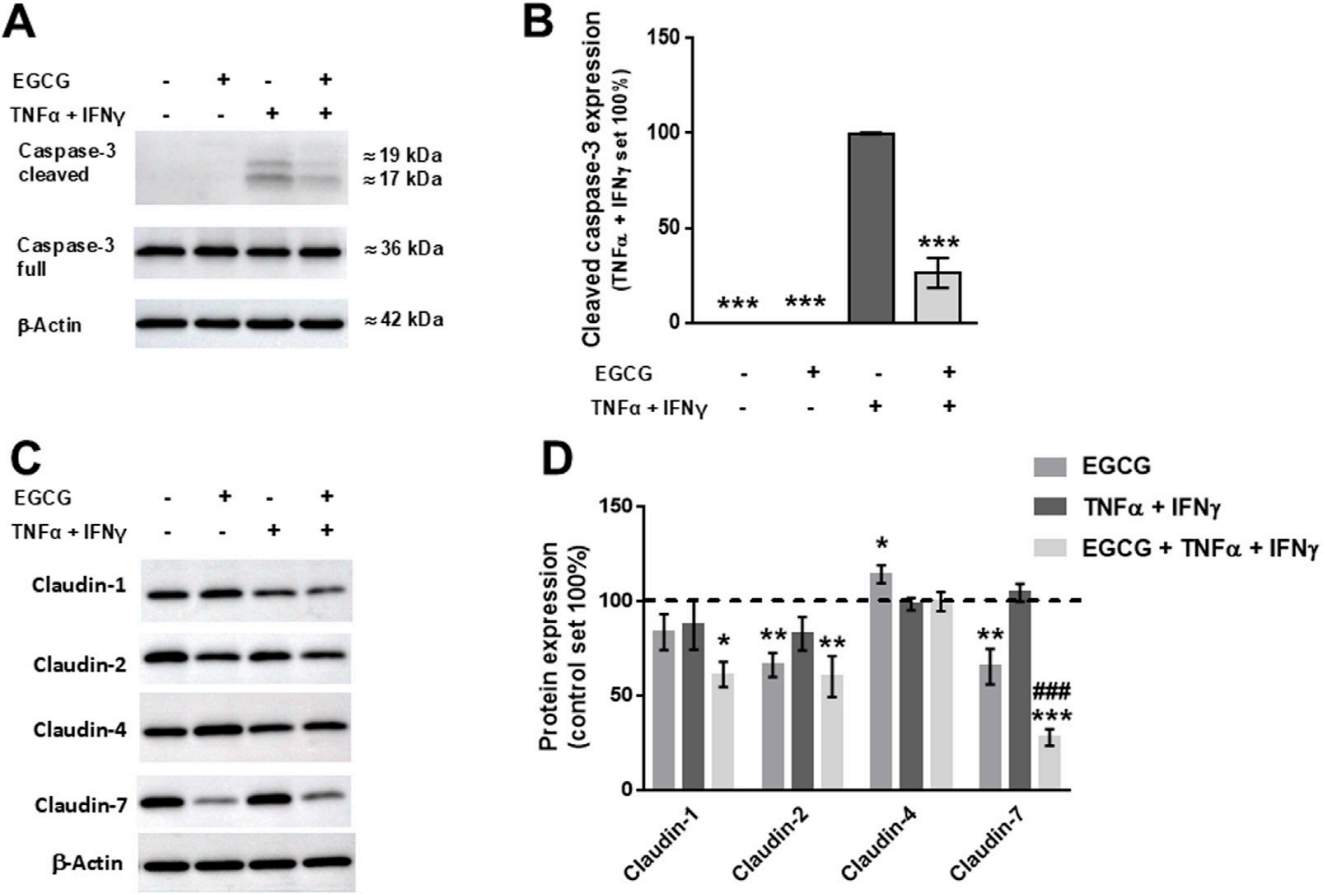
Stabilizing and protective effects of EGCG on TJ protein expression in 2D-organoids. **(A)** Representative Western blots of caspase-3 and **(B)** densitometric analysis under control conditions, incubation with EGCG (200 µM), TNFα (5 ng/mL) + IFNγ (1 ng/mL), and TNFα + IFNγ with EGCG pre-incubation. Since controls displayed no cleaved caspase-3, values of TNFα + IFNγ-treated organoids were set 100%, all values were normalized to β-actin (n = 3; ***p < 0.001 vs. TNFα + IFNγ). **(C)** Representative Western blots of TJ protein expression and **(D)** densitometric analysis. Normalization to β-actin was performed and protein expression in untreated monolayers was set 100% (n = 4–10; *p < 0.05, **p < 0.01, ***p < 0.001 vs. control, ^###^p < 0.001 TNFα + IFNγ vs. EGCG + TNFα + IFNγ).

EGCG (200 µM) increased TER on 2D-organoid monolayers by about 80% overnight (Figure 5A) and attenuated the TNFα+IFNγ-induced TER decrease, which occurred within 10 h (Figure 5B). This was accompanied by a complete inhibition of the cytokine-induced increase in FD4 permeability (Figure 5C). Consistent with the other two models, EGCG had an inhibitory effect on cytokine-induced caspase-3 cleavage (Figures 6A, B). Similar to Caco-2 monolayers, EGCG affected TJ protein expression by decreasing claudin-2 and claudin-7 and increasing claudin-4 in the 2D-organoid model. However, claudin-2 expression was not upregulated by TNFα + IFNγ (Figures 6C, D).

## 4 Discussion

Epithelial barrier function is an essential feature of a healthy gut. The present study analyzed the effects of EGCG, the main component of green tea, on intestinal barrier models under healthy conditions and after challenge with the pro-inflammatory cytokines, TNFα and IFNγ. Both cytokines are well-known to trigger intestinal barrier dysfunction in IBD. In this study, the effects of EGCG on barrier function were studied in three different human intestinal cell models. T84 cells served as a colon model while Caco-2 cells and duodenum organoid monolayers served as small intestinal models. Our study shows for the first time, that EGCG (i) affects the expression and localization of distinct barrier relevant TJ proteins and (ii) inhibits inflammatory cytokine-induced intestinal epithelial cell apoptosis. In all three models, EGCG was effective against TNFα- and IFNγ-induced barrier dysfunction. EGCG prevented the cytokine-induced decrease in TER and increase in FD4 permeability, which was related to apoptosis inhibition and changes in TJ protein expression or localization. In contrast to the colon cell line T84, EGCG additionally stabilized barrier function, as indicated by a slight but significant TER increase in unstimulated small intestinal monolayer models.

Barrier dysfunction is indicated by a TER decrease and an increase in permeability of different molecules, such as lipopolysaccharides or other antigens. The latter is usually experimentally mimicked by labeled marker molecules, such as FD4. In this study, EGCG inhibited or reduced the TNFα+IFNγ-induced decrease in TER and increase in FD4 permeability in all three models. Similar effects have been described before. In T84 cells, EGCG was shown to block a TER-decrease stimulated by IFNγ- but not by IL-4 (Watson et al., 2004). Others found a protective effect of EGCG (218 μM) against an indomethacin-induced TER decrease and increase in FD4 permeability in Caco-2 cells (Carrasco-Pozo et al., 2013). Furthermore, EGCG exerted anti-inflammatory effects in lipopolysaccharide-stimulated co-cultures of macrophages and Caco-2 cells (Schnur et al., 2023).

As shown in previous studies, TNFα and IFNγ impair epithelial barrier function by enhancing epithelial apoptosis (Bruewer et al., 2003; Kim et al., 2021; Quaranta et al., 2011) and altering TJ protein expression (reviewed in Hering et al. (2012)). In the present study, the pro-apoptotic enzyme caspase-3, which is activated by cleavage, was used as a marker of apoptosis induction. The anti-apoptotic effect of EGCG was clearly proved by the inhibition of caspase-3 cleavage in all three cell models. This is in line with a reduced FD4 permeability upon EGCG treatment. Previous studies have already suggested that this marker molecule might pass the intestinal barrier via apoptotic leaks (Hering et al., 2019; Juuti-Uusitalo et al., 2011; Krug et al., 2023). Thus, EGCG-mediated inhibition of apoptosis may contribute to its broader anti-inflammatory action. Enhanced macromolecular passage of bacterial antigens, allergens or toxins from the intestinal lumen via apoptotic leaks into the circulation (leaky gut concept) triggers systemic immune activation. This results in a vicious circle, as secreted pro-inflammatory cytokines in turn exacerbate barrier dysfunction. Since our findings suggest that EGCG interferes with this cycle, EGCG might be beneficial against numerous intestinal diseases that are associated with inflammatory barrier dysfunction, e.g. inflammatory bowel disease (reviewed in Hering et al. (2012)), chronic human immunodeficiency virus infection (Brenchley et al., 2006; Masete et al., 2025b) or graft versus host disease (Troeger et al., 2018). While recent studies on other cell types also observed apoptosis inhibition (Ge et al., 2024; Hao et al., 2024; Wei et al., 2024) others reported pro-apoptotic effects by EGCG, e.g. in colon cancer cell lines (Cerezo-Guisado et al., 2015; Shimizu et al., 2005). These seemingly conflicting results may reflect the versatile anti-carcinogenic mechanisms of EGCG, which acts via a diverse array of signaling pathways involving MAPK, EGFR, PI3K, NFκB or VEGF (Sharifi-Rad et al., 2020). Therefore, the mechanisms of action of EGCG likely depend on the distinct cell types and their physiological or immunological state of the cell, which determines the activity or responsiveness to distinct signaling pathways and receptors. Consequently, EGCG may exert distinct effects under pro-inflammatory conditions.

A recent study using a DSS colitis mouse model showed, that EGCG stimulates the mRNA expression of the TJ protein occludin and the TJ-associated scaffold protein ZO-1 (Diwan and Sharma, 2022). Our preset study proved, that EGCG also has an impact on barrier relevant TJ proteins, i.e. channel-forming or sealing TJ proteins. In T84 cells, TNFα + IFNγ caused a redistribution of claudin-1 and -5 out of the TJ. As both proteins have sealing properties (Amasheh et al., 2005; Furuse et al., 2002), their delocalization contributes to barrier loss. EGCG prevented this delocalization. In the colon cell line HT-29/B6, other phytocompounds such as berberine (Amasheh et al., 2010), myrrh (Rosenthal et al., 2017) or the ellagic acid metabolite urolithin A (Hering et al., 2021) were described to inhibit TNFα-induced redistribution of claudin-1. Claudin-5 redistribution was also found in colon biopsies of patients with Crohn’s disease (Zeissig et al., 2007) or lymphocytic colitis (Barmeyer et al., 2017) and was mimicked by TNFα and IFNγ in colon epithelial cell lines *in vitro* (Barmeyer et al., 2017). Subsequently, the protective effects of EGCG on T84 monolayers involve inhibition of cytokine-induced apoptosis and redistribution of sealing claudin-1 and -5, which is reflected by decreased FD4 permeability and higher TER values.

In contrast to T84 monolayers, EGCG altered TJ protein expression in the small intestinal cell models. EGCG reduced the expression of the channel-forming TJ protein claudin-2 which favors sodium and water passage (Amasheh et al., 2002; Rosenthal et al, 2010). Consequently, TNFα+IFNγ-induced upregulation of claudin-2 was attenuated in EGCG-treated monolayers. Claudin-2 expression is well known to be enhanced by TNFα (Mankertz et al., 2009). It is upregulated in IBD patients’ mucosa (Heller et al., 2005; Zeissig et al., 2007) and contributes to barrier leakiness. A number of phytocompounds are known to reduce TNFα-induced claudin-2 upregulation, including the ginger component 6-shogaol (Luettig et al., 2016), myrrh (Rosenthal et al., 2017) or urolithin A (Hering et al., 2021). In addition to antagonizing claudin-2’s regulation, EGCG affected the expression of claudin-4 and -7 independently of cytokine challenge in Caco-2 and organoid monolayers. Remarkably, claudin-4 and -7 levels are both downregulated in ulcerative colitis (Heller et al., 2005; Oshima et al., 2008; Stio et al., 2016). The physiological function of these two claudins is not completely clarified and seems to depend on the specific protein composition of the TJ (Günzel and Fromm, 2012). Cell culture studies on Caco-2 cells with Kampferol (Suzuki et al., 2011) or Quercetin (Amasheh et al., 2008) suggest that claudin-4 acts in a sealing manner. In knock-out mice claudin-7 was shown to be relevant for intestinal solute homeostasis and intestinal inflammation (Tanaka et al., 2015). Interestingly, claudin-7 knock-out mice showed increased claudin-4 levels, suggesting a putative co-regulation (Tanaka et al., 2015). This could also be the case in our small intestinal cell models and might explain the EGCG-induced TER increase, provided claudin-4 acts in a sealing manner. Our findings that EGCG has barrier-strengthening or -stabilizing properties are supported by other reports of EGCG-induced TER increase in Caco-2 monolayers (Li and Weigmann, 2023; Schnur et al., 2023). Taken together, in both small intestinal cell models, EGCG strengthens barrier function, probably by sealing the TJ. Moreover, it protects barrier function by antagonizing cytokine-induced epithelial apoptosis.

In the present study, EGCG improved barrier function in all three investigated models. While inhibition of cytokine-induced apoptosis seems to represent a general barrier protective mechanism in all three models, EGCG affects TJ proteins slightly differently. This might be due to a different composition of TJ proteins in these models. For the cell lines this is clearly reflected by the resistance level of the monolayers. T84 cells form high-resistance monolayers with a TER of more than 2,000 Ω*cm^2^ while Caco-2 monolayers have a much lower resistance of between 400 and 700 Ω*cm^2^. Similar to the Caco-2 monolayers, the organoid monolayers’ resistance ranged between 200 and 500 Ω cm^2^. In contrast to the epithelial cell lines, our organoid monolayers do not solely consist of epithelial cells, but also contain to a smaller extent some other cell types, paneth cells, stem cells, and goblet cells (Masete et al., 2025a). Recently we showed that our 2D organoids are a suitable model to study macromolecular transport and established a causal link between TNFα+IFNγ-induced apoptosis and epithelial permeability (Masete et al., 2025b), while former studies using cell lines reported difficulties to link these two processes (reviewed in Lechuga et al. (2023)). This could be explained by the cancerous origin of the cell lines, resulting in apoptosis resistance and aberrant signaling pathways. Compared to epithelial cell lines, the organoid monolayers might represent a more physiological model, especially for studying apoptosis-related barrier dysfunction.

Our data show that EGCG has barrier stabilizing and barrier protective effects on intestinal epithelial cells supporting its pharmaceutical application in treating intestinal diseases that are associated with barrier dysfunction such as IBD. Future studies are needed to address its underlying mechanisms in more detail, including which receptors it binds to and which signaling pathways it activates. Furthermore, the potential action of EGCG on intestinal barrier function remains to be proven in a suitable *in vivo* model. Due to fast metabolization upon oral ingestion and degradation by the gut microbiota, the bioavailability and stability of EGCG is low (Fleet et al., 2002) and high doses are probably necessary to achieve therapeutic effects (Huo et al., 2008). Some studies have shown that this can be enhanced by co-administering EGCG with different foods or by administering nanoparticle formulations, in particular polymer and lipid carriers (Andreu-Fernandez et al., 2020; Smith et al., 2010; Zhang et al., 2013). Here we identified 200 µM EGCG as an effective dose in our cell models. For comparison, in healthy volunteers an ingestion of 300 mg of green tea extract yielded a maximum blood concentration of EGCG of 5.9 ng/ml/kg for men and 6.7 ng/ml/kg for women (Andreu-Fernandez et al., 2020). According to a study of Khokhar and Magnusdottir, 1 g of dry tea leaves contains on average 67 ± 11 mg of total catechins, including about 30 mg EGCG (Khokhar and Magnusdottir, 2002). Assuming 1 g of dry tea leaves per 100 mL of water, this corresponds to a concentration of about 30 mg/100 mL. According to Wu and Wei (2002) a cup of green tea (2.5 g of green tea leaves/200 mL of water) may contain 45 mg of EGCG/100 mL of water. These differences may be due to different types of green tea, which differ in the content of catechins and EGCG (Khokhar and Magnusdottir, 2002). More research is needed to verify the effects of EGCG on barrier function in IBD patients and decipher its effective therapeutic dose.

In summary, this study demonstrates the protective effect of EGCG, the most abundant and bioactive compound in green tea, against cytokine-induced barrier damage in intestinal cell models. The study shows for the first time that EGCG (i) affects the expression and localization of distinct barrier-relevant TJ proteins, i.e., the expression of claudin-2, -4, and -7 in the small intestinal models and the localization of claudin-1 and -5 in the colon model and (ii) inhibits inflammatory cytokine-induced epithelial apoptosis in T84 cells, Caco2 cells and human intestinal 2D-organoids.

## Data Availability

The original contributions presented in the study are included in the article/Supplementary Material, further inquiries can be directed to the corresponding author.
