# Supplementary figures and images for: The green tea component (−)-epigallocatechin-3-gallate protects against cytokine-induced epithelial barrier damage in intestinal epithelial cells
Source: Front Pharmacol. 2025 May 14;16:1559812. doi: 10.3389/fphar.2025.1559812 (PMC12117334; doi:10.3389/fphar.2025.1559812)

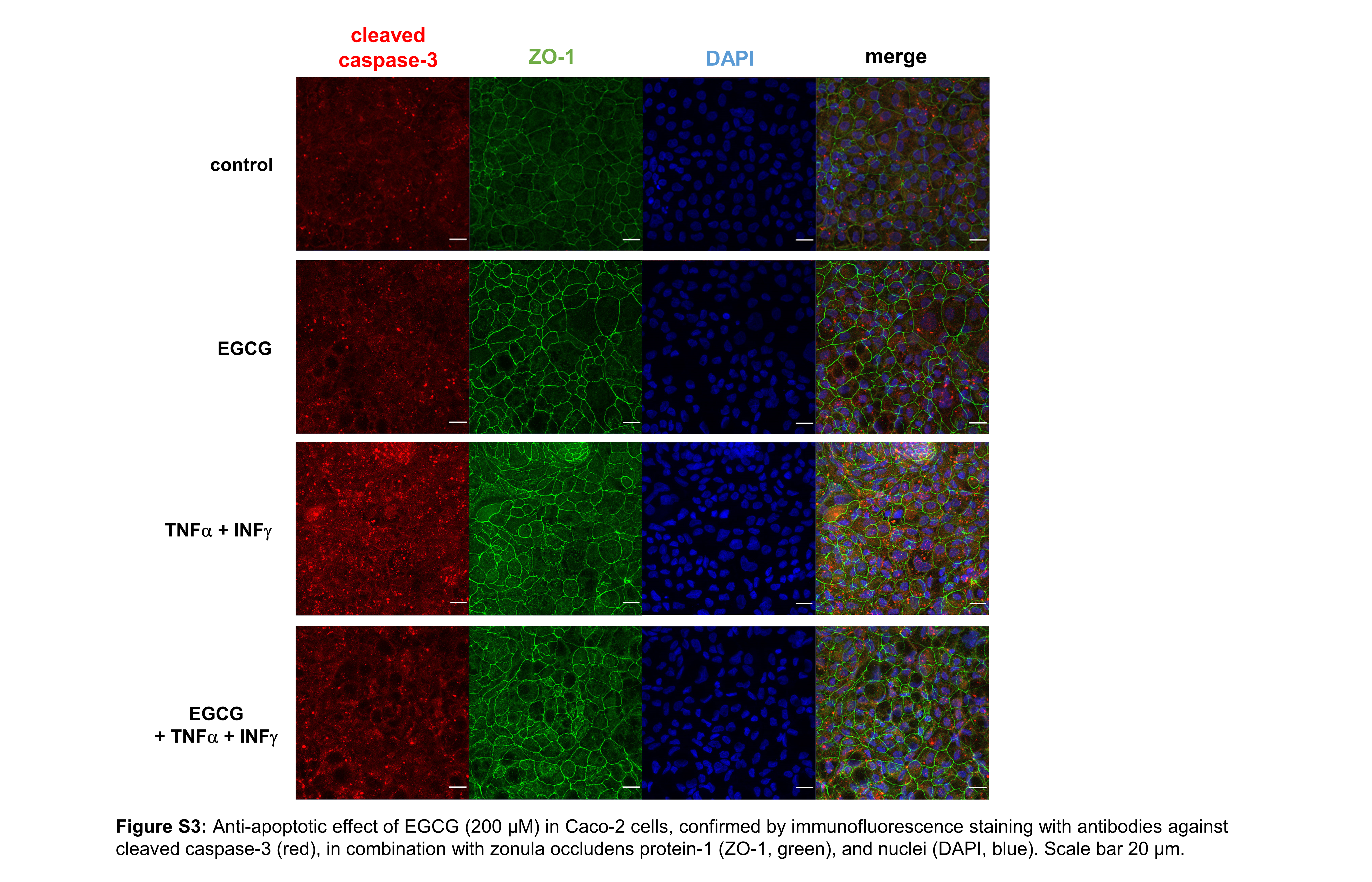

Supplement: Supplementary file 1 [file Image3.tif]

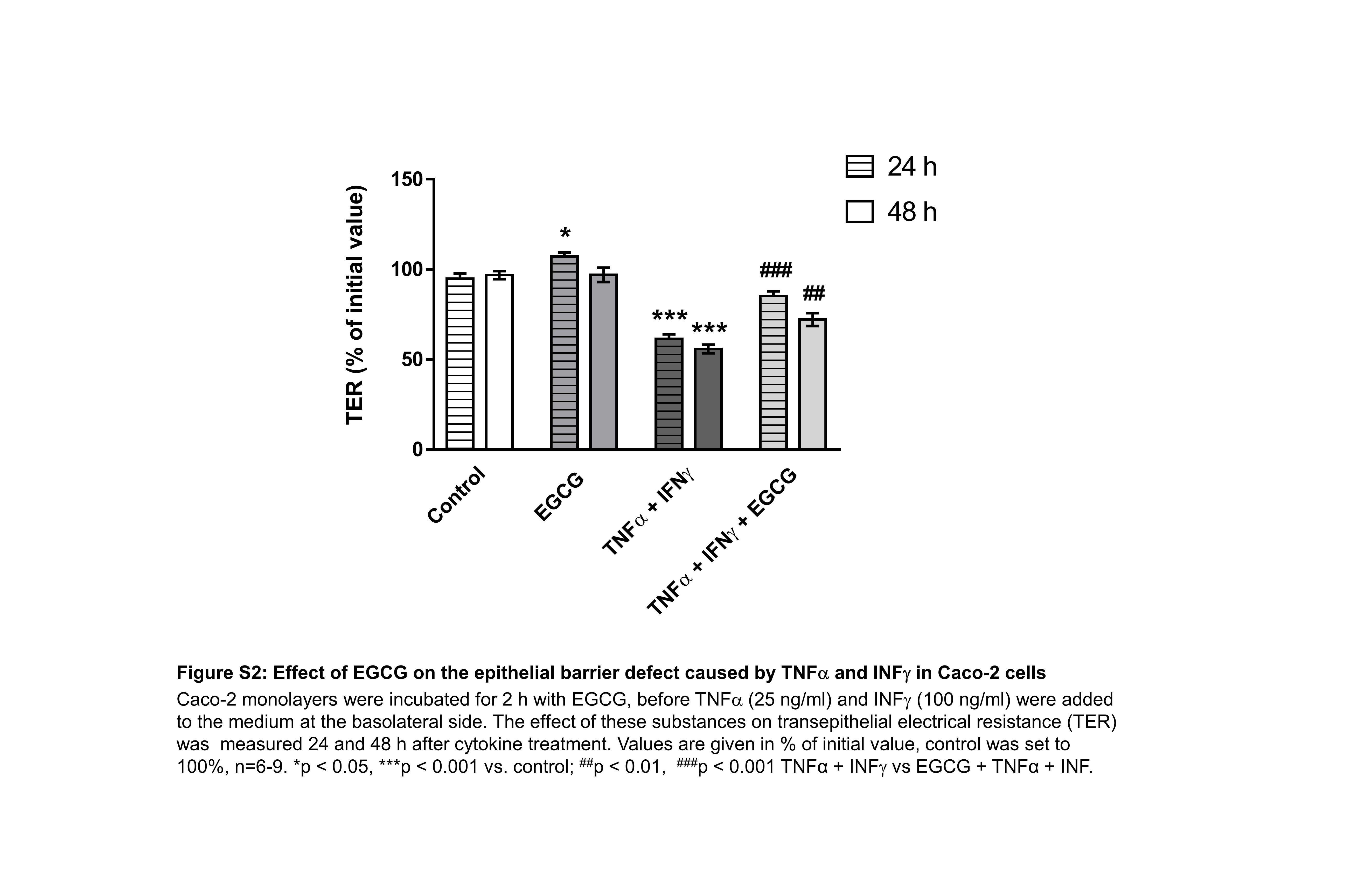

Supplement: Supplementary file 2 [file Image2.tif]

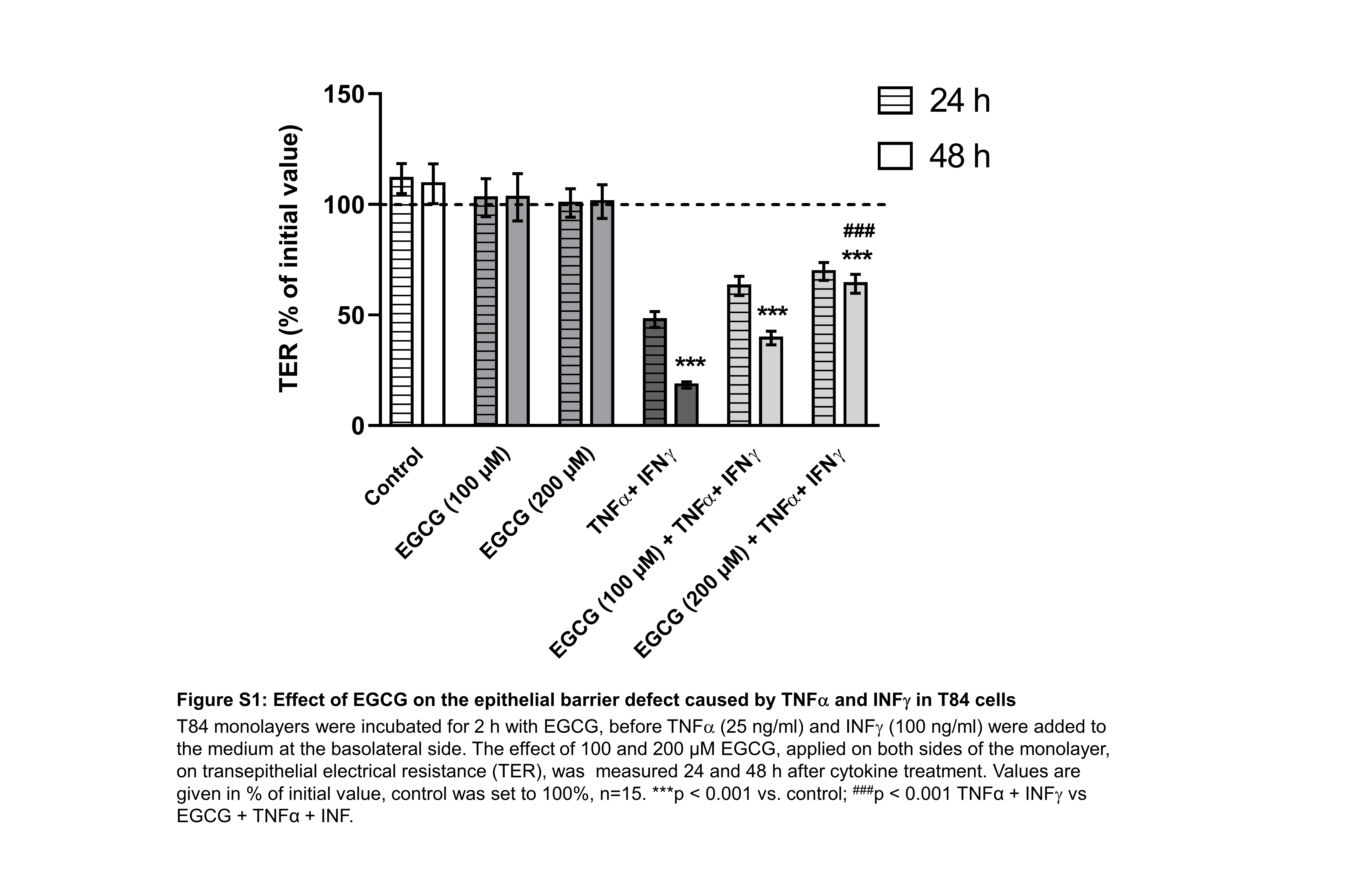

Supplement: Supplementary file 3 [file Image1.tif]
