# Supplementary material for: The green tea component (−)-epigallocatechin-3-gallate protects against cytokine-induced epithelial barrier damage in intestinal epithelial cells
Source: Front Pharmacol. 2025 May 14;16:1559812. doi: 10.3389/fphar.2025.1559812 (PMC12117334; doi:10.3389/fphar.2025.1559812)
Supplement: Supplementary file 4 [file DataSheet1.pdf]

Table S1: Primary and secondary antibodies used in the study.

**Antibodies**	**Company**	Antibody Registry ID
**Primary antibodies**		
Mouse monoclonal anti-β-actin	Sigma-Aldrich Chemie,GmbH, Steinheim, Germany	AB_476744
Rabbit monoclonal anti- β-actin	Cell Signaling Technology®, MA, USA	AB_2223172
Rabbit polyclonal anti-claudin-1	Thermo Fisher Scientific, Invitrogen, Darmstadt, Germany	AB_2533916
Mouse monoclonal anti-claudin-2	Thermo Fisher Scientific, MA, USA	AB_2533085
Rabbit polyclonal anti-claudin-2	Thermo Fisher Scientific, MA, USA	AB_2533911
Rabbit polyclonal anti-claudin-3	Abcam, Berlin, Germany	AB_301648
Rabbit monoclonal anti-claudin-4	Abcam, Berlin, Germany	AB_2732879
Rabbit polyclonal anti-claudin-5	Cell Signaling Technology®, MA, USA	AB_3065250
Rabbit polyclonal anti-claudin-7	Abcam, Berlin, Germany	AB_2783812
Rabbit polyclonal anti-claudin-8	Thermo Fisher Scientific, MA, USA	
Mouse monoclonal anti-occludin	Thermo Fisher Scientific, MA, USA	AB_2533101
Rabbit polyclonal anti-occludin	Thermo Fisher Scientific, MA, USA	AB_2533977
Rabbit polyclonal anti-tricellulin	Thermo Fisher Scientific, MA, USA	AB_10375862
Rabbit polyclonal anti-ZO-1	Thermo Fisher Scientific, IL, USA	AB_2533456
Mouse monoclonal anti-caspase-3	Cell Signaling Technology®, MA, USA	AB_2069870
Mouse monoclonal anti-cleaved caspase-3	Proteintech, Rosemont, IL, USA	AB_3665444
**Secondary antibodies**		
Peroxidase conjugated goat anti-rabbit IgG (H+L)	Jackson ImmunoResearch Laboratories, Cambridge, UK	AB_2313567
Peroxidase conjugated goat anti-mouse IgG (H+L)	Jackson ImmunoResearch Laboratories, Cambridge, UK	AB_10015289
Goat anti-mouse Alexa Fluor® 488	Thermo Fisher Scientific, MA, USA	AB_2534084
Goat anti-rabbit Alexa Fluor® 488	Thermo Fisher Scientific, MA, USA	AB_2633275
Goat anti-rabbit Alexa Fluor® 594	Thermo Fisher Scientific, MA, USA	AB_2762824
